# PARP inhibitor response is enhanced in prostate cancer when XRCC1 expression is reduced

**DOI:** 10.1093/narcan/zcaf015

**Published:** 2025-04-23

**Authors:** Kaveri Goel, Vani Venkatappa, Kimiko L Krieger, Dongquan Chen, Arun Sreekumar, Natalie R Gassman

**Affiliations:** Department of Pathology, Heersink School of Medicine, The University of Alabama at Birmingham, Birmingham, AL 35294, United States; Department of Molecular and Cellular Biology, Baylor College of Medicine, Houston, TX 77030, United States; Department of Molecular and Cellular Biology, Baylor College of Medicine, Houston, TX 77030, United States; Division of Preventive Medicine, The University of Alabama at Birmingham, Birmingham, AL 35294, United States; Department of Molecular and Cellular Biology, Baylor College of Medicine, Houston, TX 77030, United States; Department of Pathology, Heersink School of Medicine, The University of Alabama at Birmingham, Birmingham, AL 35294, United States

## Abstract

Prostate cancer (PCa) is the second most common cancer worldwide and the fifth leading cause of cancer-related deaths among men. The emergence of metastatic castration-resistant prostate cancer (mCRPC) after androgen deprivation therapy (ADT) exemplifies the complex disease management for PCa. PARP inhibitors (PARPis) are being tested to treat mCRPC in tumors with defective homologous recombination repair (HRR) to address this complexity. However, increasing resistance towards PARPi in HRR-deficient patients and the low percentage of HRR-defective mCRPC patients requires the identification of new genes whose deficiency can be exploited for PARPi treatment. XRCC1 is a DNA repair protein critical in the base excision repair (BER) and single strand break repair (SSBR) pathways. We analyzed PCa patients’ cohorts and found that XRCC1 expression varies widely, with many patients showing low XRCC1 expression. We created XRCC1 deficiency in PCa models to examine PARPi sensitivity. XRCC1 loss conferred hypersensitivity to PARPi by promoting the accumulation of DNA double-strand breaks, increasing cell-cycle arrest, and inducing apoptosis. We confirmed that XRCC1 expression correlated with PARPi sensitivity using a doxycycline-inducible system. Therefore, we conclude that XRCC1 expression level predicts response to PARPi, and the clinical utility of PARPi in PCa can extend to low XRCC1 expressing tumors.

## Graphical Abstract

**Graphical Abstract:**
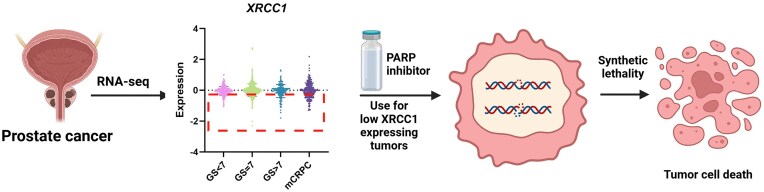

## Introduction

Prostate cancer (PCa) is the second most common cancer worldwide and the fifth leading cause of cancer-related deaths among men [1]. In the United States, PCa is the second-leading cause of cancer-related deaths, with ∼34 700 deaths estimated in 2023, representing nearly 11% of all cancer deaths in men. PCa is a clinically heterogeneous disease, ranging from highly treatable to aggressively lethal forms [2]. Androgen receptor (AR) signaling plays a central role in the development of PCa; therefore, androgen deprivation therapy (ADT) remains the first line of treatment for localized as well as advanced or metastatic PCa [3]. Resistance to ADT arises for several reasons, including but not limited to AR gain-of-function mutations or splice variants (e.g. AR-V7), AR post-translational modifications, intertumoral and adrenal androgen synthesis, glucocorticoid receptor activation, and loss of tumor suppressor genes (e.g. tumor protein 53 (p53) and phosphatase and tensin homolog (PTEN)) [4]. Castration-resistant prostate cancer (CRPC) no longer responds to ADT and is associated with poor prognosis and worse overall survival [5].

The application of PARP inhibitors (PARPis) to metastatic castration-resistant prostate cancer (mCRPC) is being tested in several clinical trials with some efficacy. The effectiveness of PARPi treatment typically depends upon the presence of defective homologous recombination (HR) repair in the tumor. In PCa, 10% of localized and 20%–30% of mCRPC harbor defects in the HR genes, offering opportunities for PARPi treatment. Synthetic lethality for PARPi in the presence of HR-deficient cells rises when the increase in double strand breals (DSB)s from the unresolved single strand breaks (SSBs) and DNA–protein complexes exceeds the ability of other repair pathways to address, promoting cell death [6]. Different PARPi, such as olaparib, rucaparib, and talazoparib, differ in their abilities to inhibit the catalytic activity of PARP1 and induce trapping of PARP1, offering different strategies for promoting cell death in HR-deficient cells [7, 8].

Several PARPi have either completed or are presently under clinical trials for treating PCa as a single agent monotherapy. For example, the PROfound study, a phase III clinical trial of olaparib, led to FDA approval for mCRPC patients who have progressed on second-generation hormone therapy and who have an identified germline or somatic gene mutation in any one of the HRR genes, including *BRCA1/2, ATM, CDK12, CHEK1, CHEK2, PALB2*, or *RAD51D* [9]. Similarly, the results of the phase III trial of rucaparib, i.e. TRITON3, led to its FDA approval for mCRPC patients harboring germline or somatic *BRCA1/2* alteration and have progressed on androgen receptor signaling inhibitors (ARSI) and taxane-based chemotherapy [10]. However, increasing resistance towards PARPi in HR-deficient patients and the low percentage of PCa patients with HR defects highlights the need for new genes or biomarkers that synergize with PARPi to create synthetic lethality [11].

In addition to HR defects, PARPi have also shown synthetic lethality with defective base excision repair (BER) [12–15]. While mapping defects in BER is less developed than in HR, several studies have shown low expression of key BER proteins increases sensitivity to PARPi. In particular, expression changes in the critical scaffold protein X-ray cross complementing protein 1 (XRCC1) are associated with hypersensitivity to PARPi in cell models and breast cancer [12, 16]. XRCC1 is a multi-domain protein with no known catalytic activity [17]. It is critical in repairing base damage and SSB via BER and single strand break repair (SSBR) pathways [18, 19]. XRCC1 is recruited by PARP1 to base lesions and strand breaks and recruits the end tailoring, gap-filling, and ligation enzymes necessary to complete repair [17].

Low XRCC1 mRNA and protein levels in cancer cells are associated with increased cancer risk, poorer survival, increased tumor aggressiveness, and resistance to some therapies [20, 21]. We recently examined XRCC1 protein levels in a tissue microarray containing benign adjacent and PCa tissues and noted a wide variance in XRCC1 protein expression across PCa tumors [22], with significantly lower expression in African-American (AA) PCa. Importantly, AA PCa patients are reported to have aggressive PCa with poor clinical outcomes. They are also resistant to many of the standard-of-care regimens, necessitating the identification of new treatment strategies. Given that low *XRCC1* gene expression has also been shown to enhance the cytotoxic effects of PARPi in breast cancer cell lines, we hypothesized that XRCC1 deficiency could be a promising target for treatment with PARPi in PCa and mCRPC [16], especially for AA PCa patients. In this work, as a proof-of-principle, we generated XRCC1 deficient PCa cell lines to test the clinical utility of PARPi beyond HR genes. Our data concludes that XRCC1 deficiency hypersensitizes PCa cells to multiple PARPi, including olaparib, rucaparib, and talazoparib.

## Materials and methods

### Cell lines culture

The PCa cell lines 22RV1, C4-2B, LNCaP, PC3, MDA-PCA-2B, and the human embryonic kidney cell line HEK293T were obtained from American Type Cell Culture (ATCC). 22RV1, C4-2B, LNCaP, and PC3were cultured in 10% fetal bovine serum (FBS, Gibco, Waltham, MA, USA) supplemented RPMI 1640 (Corning, Corning, NY, USA). The MDA-PCA-2B were cultured in 20% FBS supplemented HPC1 (Athena Enzyme Systems, Baltimore, MD, USA). The HEK293T were cultured in DMEM (Hyclone) with 10% FBS. Cells were grown in 5% CO_2_ at 37°C. The cell lines were typically cultured no longer than 20 passages upon thawing the frozen vials and were tested each month for *Mycoplasma* contamination using MycoAlert^®^ Mycoplasma Detection Kit (Lonza, Walkersville, MD, USA).

### Plasmids and constructs

The *XRCC1*-targeted guide RNA (gRNA) (GAATGATGGCTCAGCTTTCG) containing XRCC1 CRISPR gRNA2_pLentiCRISPR v2 and the only pLentiCRISPR v2 used to prepare the vector control cells were purchased from GenScript (Piscataway, NJ, USA). *XRCC1* CDS-containing overexpression plasmid (vector ID: VB230413-1287czz) was purchased from Vector Builder (Chicago, IL, USA). This construct contains mutated PAMs to prevent its targeting from gRNA. Doxycyline-inducible *XRCC1* CDS-overexpressing plasmid was generated in our lab by subcloning the PAM mutated *XRCC1* CDS into the vector pCW57-MCS1-P2AMCS2 (blasticidin) between EcoR1 and Mlu1 restriction sites. The vector pCW57-MCS1-P2AMCS2 (Blast) was a gift from Adam Karpf (Addgene plasmid #80921). The subcloning was performed by using the following primers: forward primer: GCATGCGAATTCATGCCGGAGATCCGCCTCC and reverse primer: TATCGACGCGTTCAGGCTTGCGGCACCACCC. The lentiviral packaging plasmids psPAX2 and pMD2.G were obtained from Dr. Marie Migaud (University of South Alabama).

### Lentiviral packaging

For lentiviral particle formation, 1.2 × 10^6^ HEK293T cells were seeded into 60-mm dishes. The cells were transfected after 16–20 h of seeding with the plasmids of the lentiviral packaging mix (psPAX2 and pMD2.G), along with the transfer plasmid using FuGENE^®^ 6 Transfection Reagent (Promega, Fitchburg, WI, USA), as per manufacturer’s instructions. The three plasmids were used in the following amounts: 3 μg of transfer plasmid, 2.25 μg of psPAX2 and 0.75 μg of pMD2.G. The media of cells was safely replaced with the fresh medium after 16 h of transfection. The desired viral particles containing media were then harvested after 48 h of replacement with fresh media. The collected media was centrifuged at 1000 rpm for 5 min, aliquoted into smaller volumes, and kept at −80°C until further use.

### Stable cell lines generation

For generating stable lines, C4-2B (0.5 × 10^6^) and 22RV1 (1 × 10^6^) cells were infected with 500 μl of the lentiviral particles along with 8 μg/ml polybrene (Sigma–Aldrich, Burlington, MA, USA) in 60-mm dishes at the time of seeding. Culture media was changed after 48 h, and antibiotic selection was started 2 days post-infection. 2 μg/ml puromycin (#A11138-03; Gibco) was used to select both the C4-2B and 22RV1 cells. 2 μg/ml and 3 μg/ml blasticidin S (#A11139-03; Gibco) was used to select C4-2B and 22RV1 cells, respectively. For generation of *XRCC1* knockout monoclones, 500 cells from the polyclonal population were seeded into 100-mm dishes and allowed to grow for the next 15–20 days. The colonies were then collected using a sterile tweezer and cloning discs and transferred to a 12-well plate. The monoclones were propagated by culturing gradually into bigger volumes in antibiotic-free media. The monoclones were screened and confirmed by immunoblotting and DNA sequencing for *XRCC1* knockout. For DNA sequencing, the *XRCC1* genomic region flanking the gRNA target site was first amplified by using the following primers: forward primer: GGTGACATGTGCTGTTTGTCT and reverse primer: CCTAGATTTTAGCCCAGTGAGAGTG. The amplicon was then sequenced by utilizing the following primer: CTGCTCCATGCCATACCCT.

### Cell growth inhibition assay

Cytotoxicity was determined with a cell growth inhibition assay. 10 000 cells per well were seeded in 1 ml media into a 24-well plate. Cells were treated after attaching for 48 h. Cells were treated with increasing concentrations of PARPi, olaparib (#S1060; Selleck chemicals, Pittsburgh, PA, USA), rucaparib (#S4948; Selleck chemicals), and talazoparib (#NC2227311; Selleck chemicals). A 50 mM stock of olaparib and rucaparib and 10 mM stock of talazoparib were prepared in dimethyl sulfoxide (DMSO, #MT-25950CQC; Corning). The final drug concentrations were prepared by dilution in the growth medium. The cells were kept in the drug-containing media for the next 6 days. Additionally, cells were treated with increasing doses of the alkylating agent methyl methanesulfonate (MMS) (#129 925; Sigma–Aldrich) to confirm BER deficiency. A 100 mM stock of MMS was diluted in growth medium and used to prepare the final lower dosing concentrations. Cells were exposed to MMS-containing media for 1 h, after which the cells were washed with PBS and incubated for the next 6 days in fresh MMS-free medium. The cells were counted with a TC20 automated cell counter (Bio-Rad, Hercules, CA, USA). The percentage survival was calculated for each cell line by normalizing the cell number obtained at a particular drug concentration to the cell number obtained at their respective DMSO vehicle control or medium control condition. Dose–response curves were prepared using the average of three biological replicates to generate the half-maximal inhibitory concentration (IC_50_) values using a nonlinear regression model in GraphPad Prism software. Values are presented as mean ± standard error of the mean (SEM).

### Clonogenic assay

C4-2B (0.5 × 10^3^) and 22RV1 (1 × 10^3^) cells were seeded in 2 ml media per well into a 12-well plate. C4-2B and 22RV1 were treated with PARPi or DMSO vehicle control after 48 h of attachment. 0.5 μM rucaparib and 0.5 nM talazoparib were used to dose both C4-2B and 22RV1. Olaparib was used at a concentration of 0.25 μM for C4-2B and 2.5 μM for 22RV1. The cells were grown in the same drug-containing media for 15 days. The plate was removed from the incubator, and the culture medium was aspirated. Cells were fixed with 3.7% formaldehyde (Thermo Fisher Scientific) in PBS for 10 min. After the fixation, plates were stained with crystal violet solution (0.1% w/v) in methanol for 10 min at room temperature (RT). The staining solution was removed, and the wells were rinsed twice with double-distilled water (ddH_2_O). The plate was allowed to dry overnight at RT. The stained plates were imaged with a ChemiDoc XRS Imaging system (Bio-Rad).

### Immunofluorescence

22RV1 cells (1 × 10^4^) were seeded in an 8-well chamber, and C4-2B cells (2 × 10^4^) were seeded on coverslips in 24-well plate. The cells were allowed to adhere for 48 h at 37°C in a 5% CO_2_ incubator. C4-2B and 22RV1 were treated with PARPi or DMSO vehicle control for 48 h. 0.5 μM rucaparib and 0.5 nM talazoparib were used to treat C4-2B and 22RV1. Olaparib was used at a concentration of 0.25 μM for C4-2B and 2.5 μM for 22RV1. Cells were fixed in 3.7% formaldehyde in PBS for 10 min at RT and then washed three times with PBS. Cells were permeabilized with permeabilization buffer (Biotum, Fremont, CA, USA) for 10 min at RT, followed by washing three times with PBS. Cells were then blocked with 2% bovine serum albumin (BSA) in PBS for 30 min at RT. 1:500 diluted phospho-Histone H2A.X (Ser139) antibody (#9718; CST), prepared in 2% BSA in PBS, was added to the cells and incubated for 1 h at RT. The excess antibody was removed by washing with PBS three times, 5 min for each wash. 1:2000 diluted anti-rabbit Alexa Fluor 647 (#A21247; Thermo Fisher Scientific), prepared in 2% BSA in PBS, was added for 1 h at RT. Nuclear DNA was stained using 1:1000 Hoechst 33 342 (#62249; Thermo Fisher Scientific) solution prepared in 2% BSA in PBS for 10 min before the end of the incubation of the secondary antibody. The cells were washed three times in PBS, 5 min for each wash. The 22RV1 cells were left in PBS at 4°C until imaging. The C4-2B cells-containing coverslips were mounted on glass slides using ProLong^™^ Gold Antifade Mountant (#36930; Thermo Fisher Scientific). Slides were allowed to dry overnight in the dark at RT and stored at 4°C until analysis. Images were acquired using a Keyence BZ-X fluorescence microscope (Osaka, Japan) with a ×60 air immersion objective. Nikon NIS-Elements Image Analysis software exported and analyzed the acquired image data. 3–4 random frames were analyzed to obtain a minimum of 100 cells per condition, and then the total number of cells in each frame and the cells which contain γH2AX foci were counted. The results were expressed as the percentage of cells that contain γH2AX foci.

### Immunoblotting

C4-2B (0.4 × 10^6^) and 22RV1 (8 × 10^6^) cells were seeded in 60-mm dishes in 4 ml media and attached for 48 h. Following attachment, cells were treated with 0.5 μM rucaparib or DMSO vehicle control for 48 h. The cells’ culture medium was aspirated, and cells were scraped using PBS and then pelleted. The cells were lysed with ice-cold lysis buffer containing β-glycerophosphate, Tris–HCl, NaCl, 0.2% Triton X-100, 0.3% NP-40, plus Halt^™^ protease and phosphatase inhibitor cocktail (#78441; ThermoFisher Scientific). Cells were incubated on ice for 30 min, centrifuged at 11 000 rpm for 15 min at 4°C, and the supernatant fraction was collected. Protein concentration was determined via the Quick Start Bradford 1× Dye Reagent (#5000205; Bio-Rad) and bovine serum albumin standard prediluted set (#23208; ThermoFisher Scientific). 25 μg of protein was separated on 4%–15% Mini-Protein TGX precast gel (Bio-Rad) ran at 120 V for 1 h. The gel was then transferred to a nitrocellulose membrane (Bio-Rad) and blocked for 1 h at RT in 5% skim milk, prepared in tris-buffered saline with 0.1% Tween 20 (TBST). The membrane was incubated overnight at 4 °C with the primary antibodies detailed in Table 1.

**Table 1.** Antibodies used for immunoblotting experiments

Antibody	Dilution	Source
XRCC1 (#MA5-12071)	1:1000	Invitrogen, Waltham, MA, USA
ATM (#2873)	1:1000	Cell Signaling Technology, Danvers, MA, USA
ATR (#2790)	1:1000	Cell Signaling Technology
pATM-S1981 (#5883)	1:500	Cell Signaling Technology
pATR-S428 (#2853)	1:500	Cell Signaling Technology
CHK1 (#2360)	1:1000	Cell Signaling Technology
CHK2 (#6334)	1:1000	Cell Signaling Technology
pCHK1-S345 (#2348)	1:500	Cell Signaling Technology
pCHK2-T68 (#2197)	1:500	Cell Signaling Technology
PARP1 (# 556494)	1:2000	BD Biosciences, Franklin Lakes, NJ, USA
Cleaved PARP1-D214 (#5625)	1:500	Cell Signaling Technology
Cleaved caspase 3-D175 (#9661)	1:500	Cell Signaling Technology
Caspase3 (#GTX13585)	1:500	GeneTex, Irvine, CA, USA
Cyclin B1 (#12 231)	1:1000	Cell Signaling Technology
p21 (#sc-397)	1:500	Santa Cruz, Dallas, TX, USA
β-Actin (#AM4302)	1:5000	Invitrogen
α-Tubulin (#T9028)	1:5000	Sigma–Aldrich, Burlington, MA, USA
H3 (#14269)	1:5000	Cell Signaling Technology

Subsequently, the membrane was washed three times with TBST for 5 min each time. HRP-conjugated 1:5000 diluted anti-mouse (#7076; CST) or anti-rabbit (#7074; CST) secondary antibody prepared in 5% skim milk in TBST was added for 1 h. The membrane was again washed three times with TBST for 5 min each time. The signals were visualized using enhanced chemiluminescence (Advansta, San Jose, CA, USA) and the ChemiDoc XRS Imaging system (Bio-Rad). For all immunoblot experiments, β-actin was used as a loading control. Immunoblots were conducted in triplicates with a representative blot shown in the figures. Quantification of the blots was done using Imagelab software (Bio-Rad). Values are presented as mean ± standard error of the mean (SEM).

### Chromatin fractionation

C4-2B (0.4 × 10^6^) and 22RV1 (8 × 10^6^) cells were seeded in 60-mm dishes in 4 ml media and attached for 48 h. Following attachment, cells were treated with 2.5 μM rucaparib or DMSO vehicle control for 24 h. The cells’ culture medium was aspirated, and cells were scraped using phosphate buffered saline (PBS) and then pelleted. The cells were resuspended in lysis buffer containing 30 mM Tris–HCl pH 7.5, 150 mM NaCl, 0.5% Triton X-100, 2 mM MgCl_2_ plus Halt^™^ protease and phosphatase inhibitor cocktail. The cell suspensions were incubated on ice for 15 min followed by centrifugation for 15 min at 15 000 × *g* at 4 °C to pellet the chromatin. The supernatant was used as a soluble fraction. The pellet was washed three times with lysis buffer. The sample was vortexed for each washing step, and chromatin was pelleted for 5 min at 15 000 × *g* at 4 °C. The chromatin pellet was dissolved in a solution containing 8 mM NaOH and 1% sodium dodecyl sulfate (SDS). All samples were boiled at 95 °C for 15 min after adding 2× Laemmli Sample Buffer (#1610737; Bio-Rad). Samples were centrifuged for 15 min at 15 000 × *g* to collect the supernatant and subjected to immunoblotting. α-tubulin and H3 were used as a loading control for soluble and chromatin fractions, respectively. Immunoblots were conducted in triplicates, with a representative blot shown in the figures. Quantification of the blots was done using Imagelab software. Values are presented as mean ± standard error of the mean (SEM).

### Cell cycle analysis

For cell cycle analysis, C4-2B (0.7 × 10^6^) and 22RV1 (1.5 × 10^6^) cells were seeded in 100-mm dishes in 10 ml media and allowed to attach for 48 h. Following attachment, cells were treated with 0.5 μM rucaparib or DMSO vehicle control for 48 h. 1 × 10^6^ cells per condition were collected after trypsinization in 15 ml tubes, washed twice in PBS, and fixed in 10 ml ice-cold 70% ethanol, prepared in ddH_2_O at −20°C overnight. Cells were pelleted at 1000 rpm for 5 min and washed with PBS twice. Cells were incubated in 20 μg/ml propidium iodide (PI) (#P3566; Invitrogen) and 10 μg/ml RNAase (#EN0531; Thermo Fisher Scientific) containing 500 μl PBS for 20 min at 37°C in the dark. Samples were run on BD LSRFortessa^™^ X-20 cell analyzer (BD Biosciences, Franklin Lakes, New Jersey, USA). 10 000 events were collected for each sample, and the data was analyzed in FlowJo version 10.7 using the in-built Watson Pragmatic algorithm of the cell cycle.

### Cell death assay

Cell death was measured using the Annexin/PI Apoptosis Detection Kit (#A432; Leinco Technologies, St. Louis, MO). C4-2B (0.3 × 10^6^) and 22RV1 (0.4 × 10^6^) cells were seeded in 60-mm dishes in 4 ml media and allowed to attach for 48 h. Following attachment, cells were treated with 0.5 μM rucaparib or DMSO vehicle control for 48 h. The cells’ culture media was then collected. Cells were washed with PBS, trypsinized, and added to their respective media-containing tubes. The cells were pelleted by centrifugation at 1000 rpm for 5 min and resuspended in ice-cold PBS. 0.25 × 10^6^ cells per condition were collected and pelleted again. 300 μl of binding buffer along with 5 μl of annexin and 5 μl of PI were added to the cells pellet, and cells were then resuspended and incubated at RT in the dark for 15 min. Samples were run on BD LSRFortessa^™^ X-20 cell analyzer. 10 000 events were collected for each sample, and the data were analyzed in FlowJo version 10.7. Annexin positive and PI negative were classified as early-stage apoptotic cells, annexin positive and PI positive were classified as late apoptotic cells, and PI-stained cells were classified as necrotic cells.

### Doxycycline induction

Doxycyline-inducible *XRCC1* CDS-overexpressing *XRCC1-*KO C-1 C4-2B cells (0.2 × 10^6^) were seeded per well in 3 ml media in a 6-well plate and attached for 48 h. The cells were then treated with different concentrations of doxycycline. A 10 mg/ml stock of doxycycline was prepared in sterile water (#D9891; Sigma). The final doxycycline concentrations were prepared by diluting the stock in the growth medium. The cells were kept in the doxycycline-containing media for the next 24 h, after which they were harvested and used for immunoblotting to detect *XRCC1* expression level. Only media was used for control cells.

For performing cell growth inhibition assay in the same cells, 10 000 cells were seeded per well in 1 ml media in a 48-well plate and allowed to attach for 48 h. The cells were exposed to different concentrations of doxycycline-containing media for 24 h in the same way as mentioned above. Following 24 h, the doxycycline-containing media of the cells was spiked with rucaparib to obtain a final concentration of 10 μM. The cells were kept in the doxycycline and rucaparib-containing media for the next 72 h, followed by cell counting.

### Statistical analysis

Two-way ANOVA determined statistical significance with Tukey’s multiple comparisons test using GraphPad Prism version 10. The differences between the groups were considered significant if the *P*-value was ≤ 0.05. Statistical significance was defined by *P ≤ 0.05, **P ≤ 0.01, ***P ≤ 0.001, and ****P ≤ 0.0001. The error bars represent the standard error of the mean (SEM) obtained from three independent experiments.

## Results

### PCa patients exhibit a wide range of *XRCC1* expression

**Figure 1.**
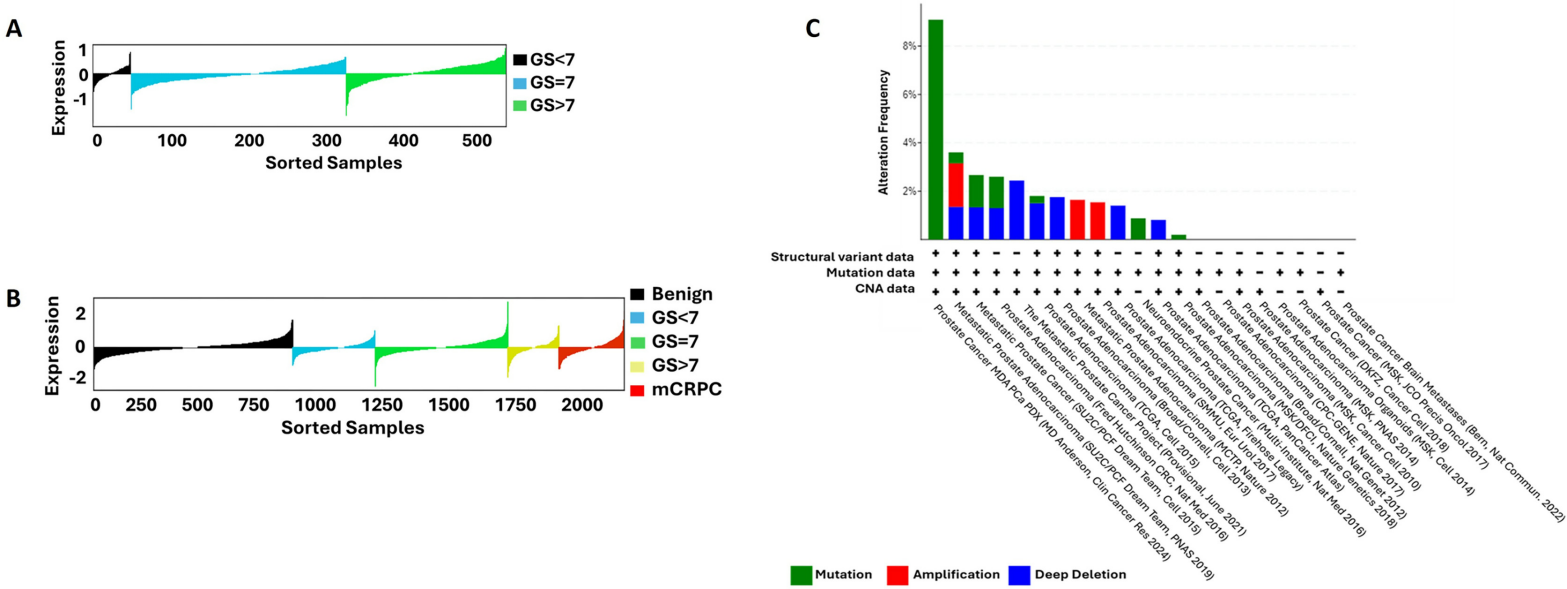
Expression level and alteration status of *XRCC1* in PCa patients. (**A**) Lollipop plot of *XRCC1* Log2 median-centered and quantile-scaled normalized gene expression values from TCGA in benign prostate, PCa, and mCRPC patient samples. (**B**) Lollipop of *XRCC1* Log2 median-centered and quantile scaled normalized gene expression values from PCTA in benign prostate, PCa, and mCRPC patient samples. The plots in panels (A) and (B) were generated from the website (http://www.thepcta.org/); GS: Gleason score. (**C**) A bar diagram showing the frequency and type of alterations in the *XRCC1* gene among PCa patients from different cohorts was generated using cBioportal website (https://www.cbioportal.org/).

To confirm mRNA expression levels of XRCC1 varied within PCa tumors, we examined *XRCC1* mRNA expression from The Cancer Genome Atlas (TCGA) and The Prostate Cancer Transcriptome Atlas (PCTA) databases (http://www.thepcta.org/) [23]. Both datasets indicate that while the mean mRNA expression of *XRCC1* doesn’t significantly change between benign, primary, and metastatic PCa, a wide range of expression levels are observed in patients across these categories, with both databases demonstrating a significant number of patients expressing low mRNA levels of *XRCC1* (Fig. 1A and B). We also examined cbioportal (https://www.cbioportal.org/) and noted that several PCa cohorts showed *XRCC1* deletion with a maximum deletion frequency of 2.44% (Fig. 1C) [24–26]. Additionally, there is a low frequency of XRCC1 mutations (0.5%–8%) across the patient cohorts. Therefore, we hypothesized that XRCC1 deficiency could be a potential biomarker for PARPi treatment across different PCa stages.

### *XRCC1* depletion enhances the sensitivity of PCa cells to PARPis

**Figure 2.**
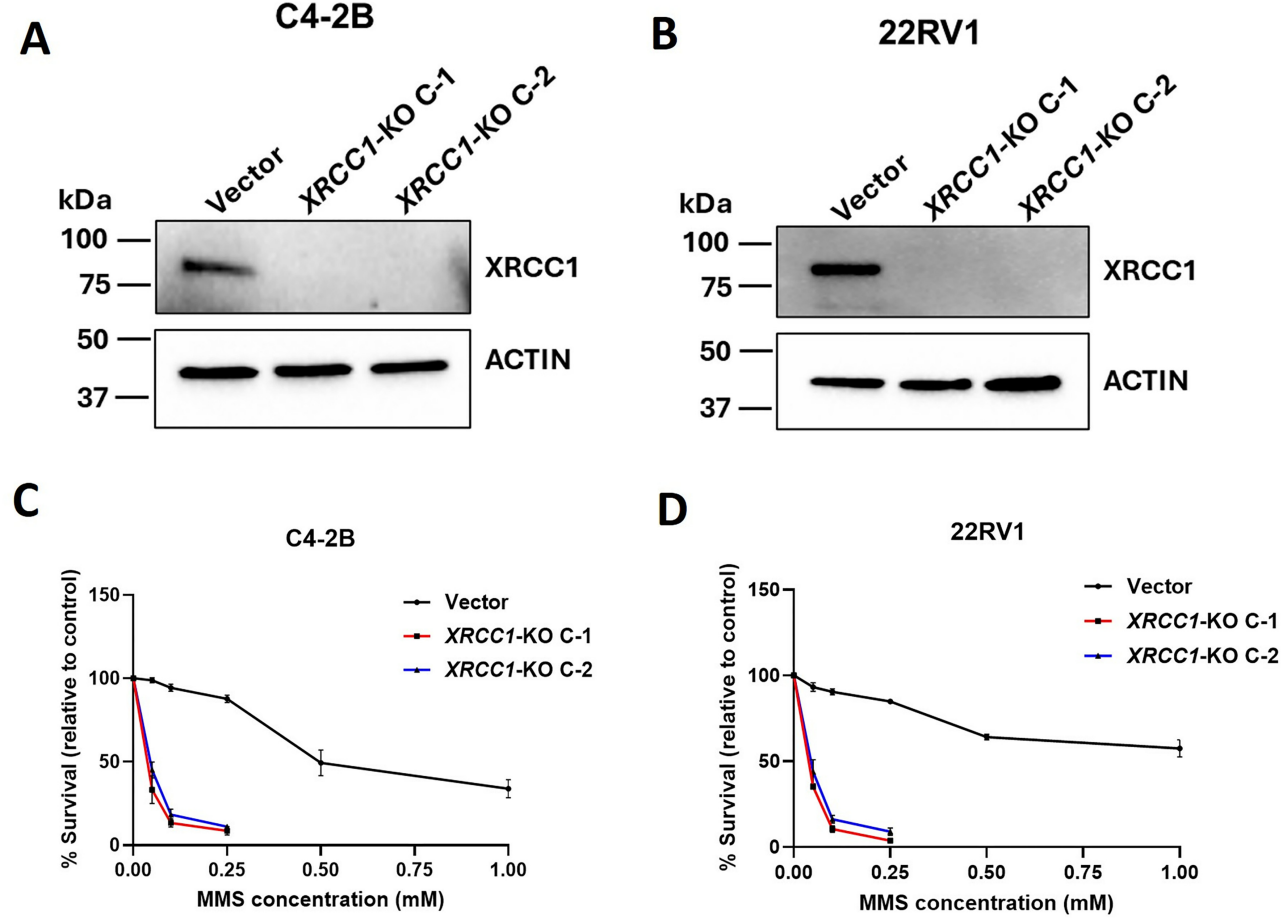
Generation of *XRCC*1 knockout PCa cell lines and their functional validation. (**A** and **B**) Immunoblot showing the expression of XRCC1 protein in C4-2B (A) and 22RV1 (B) vector control cells, *XRCC1-*KO C-1, and *XRCC1-*KO C-2. Actin was used as a loading control. (**C** and **D**) Cell growth inhibition assay of the same cells as in panels (A) and (B) with increasing concentrations of MMS. The cells were incubated in the drug-containing media for 1 h, after which the fresh drug-free media was added, and the cells were allowed to grow for 6 days. The data represent the average % survival relative to control cells ± SEM of three biologically independent samples.

We generated *XRCC1*-deficient stable cell lines using CRISPR/Cas9 in two mCRPC PCa cell models: C4-2B and 22RV1. We isolated two *XRCC1-*KO monoclones, *XRCC1-*KO C-1 and *XRCC1-*KO C-2, for each cell line. We then performed polymerase chain reaction (PCR) to amplify the gRNA target sequence-surrounding region utilizing gRNA target site flanking primers from the vector control and *XRCC1*-KO C-1 and C-2 cell lines. The amplicons were then sequenced and checked by sequence alignment on the program Clustal Omega (https://www.ebi.ac.uk/jdispatcher/msa/clustalo). Supplementary Fig. S1A shows the location of the gRNA target sequence on the *XRCC1* gene and the location of primers used for PCR and sequencing. The sequence alignment results showed a 100% alignment of amplicons from C4-2B and 22RV1 *XRCC1-*KO C-1 and *XRCC1-*KO C-2 cell lines up to the Cas9 target site, beyond which the region is frameshifted (Supplementary Fig. S1A–E). The loss of XRCC1 expression in *XRCC1-*KO C-1 and *XRCC1-*KO C-2 for each cell line was confirmed at the protein level by immunoblotting (Fig. 2A and B). We further validated the loss of function of XRCC1 in *XRCC1-*KO C-1 and *XRCC1-*KO C-2 cell lines by checking their sensitivity towards the alkylating agent, MMS. In earlier studies, XRCC1 protected mouse and human cells from MMS-induced DNA damage [27, 28]. As expected, *XRCC1-*KO C-1 and C-2 for each cell line showed hypersensitivity towards MMS, resulting in almost a 70%–80% decrease in cell viability compared with their respective vector control cells (Fig. 2C and D).

**Figure 3.**
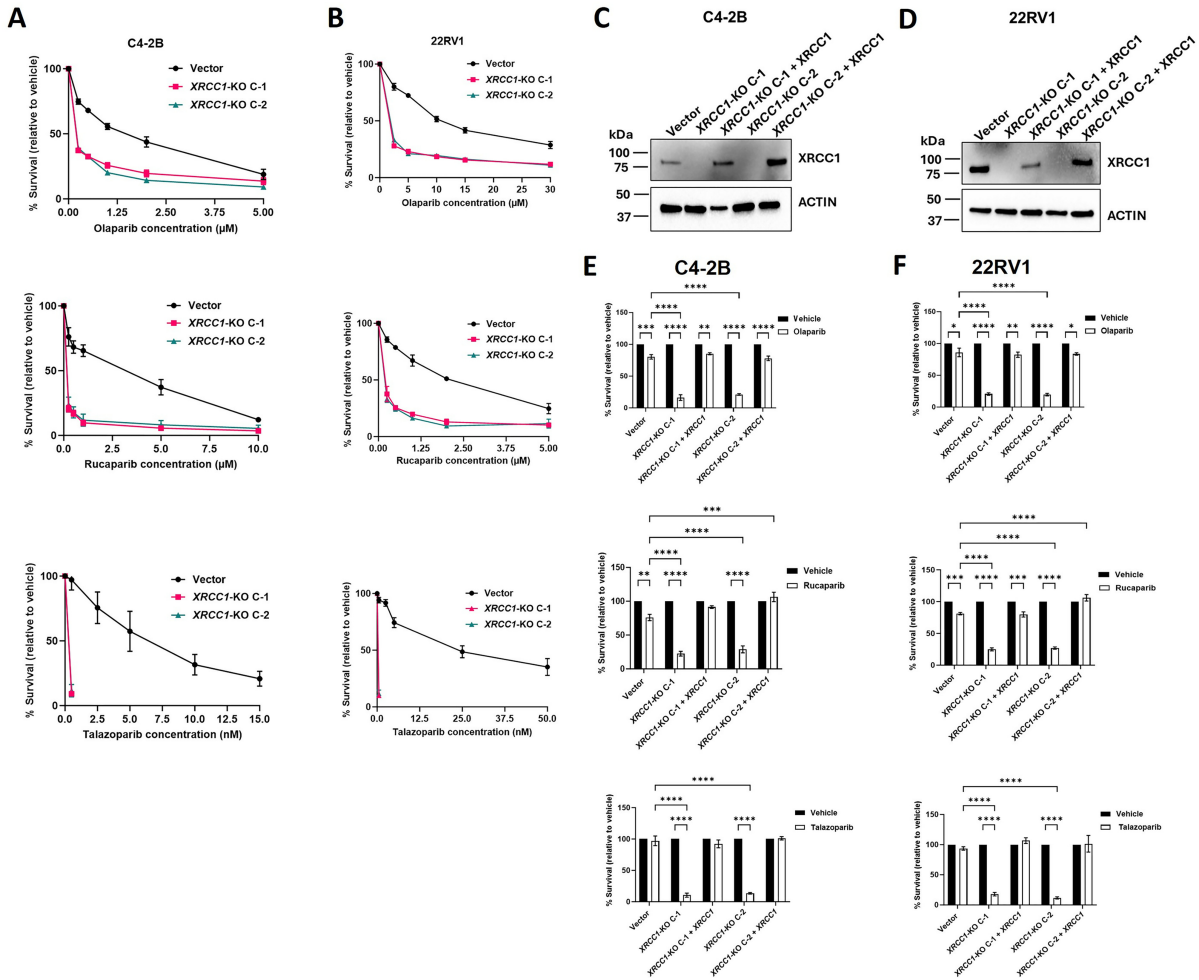
*XRCC1* depletion enhanced the sensitivity of PCa cells to PARPi. (**A** and **B**) Cell growth inhibition assay for C4-2B (A) and 22RV1 (B) vector control cells, *XRCC1-*KO-C1, and *XRCC1-*KO-C2 with increasing concentrations of PARPi: olaparib (upper), rucaparib (middle), and talazoparib (lower). The cells were incubated in the drug-containing media for 6 days. (**C** and **D**) Immunoblot showing the expression of XRCC1 protein in C4-2B (C) and 22RV1 (D) vector control, *XRCC1-*KO C-1, *XRCC1-*KO C-1 + *XRCC1*, *XRCC1-*KO C-2, and *XRCC1-*KO C-2 + *XRCC1* cells. Actin was used as a loading control. (**E** and **F**) The survival percentage of the same cells was calculated after treatment with 0.25 μM (for C4-2B) and 2.5 μM (for 22RV1) olaparib (upper), 0.5 μM rucaparib (middle), and 0.5 nM talazoparib (lower) for 6 days. The data represents the average % survival relative to the control ± SEM of three biologically independent samples. The level of statistical significance among the groups was computed using two-way ANOVA with Tukey’s multiple comparisons test. The level of statistical significance is indicated as follows: *P < 0.05, **P < 0.01, ***P < 0.001, and ****P < 0.0001.

We then assessed the susceptibility of C4-2B and 22RV1 *XRCC1-*KO lines to varying concentrations of three different PARPi: olaparib, rucaparib, and talazoparib. As expected, the vector control cell lines showed resistance to all three PARPi used, with olaparib and rucaparib showing IC_50_ values in the micromolar range. The vector control cell lines were more sensitive to talazoparib, with nanomolar IC_50_ values (Fig. 3A–C and Table 2). The C4-2B and 22RV1 *XRCC1-*KO C-1 and C-2 showed at least a 10-fold decrease in IC_50_ value of olaparib and 20-fold (in C4-2B) and 30-fold (in 22RV1) decrease in IC_50_ value of rucaparib compared to their respective vector controls (Fig. 3A–C and Table 2). The *XRCC1-*KO C-1 and C-2 showed even greater sensitivity to talazoparib with 0.5 nM doses, resulting in an ∼90% reduction in cell viability.

**Table 2.** IC_50_ values of PARPi in the mCRPC cell lines with XRCC1 depletion

Cell line	Olaparib IC_50_ (μM) ± SEM	Rucaparib IC_50_ (μM) ± SEM	Talazoparib IC_50_ (nM) ± SEM
C4-2B vector	1.35 ± 0.23	3.01 ± 0.17	5.43 ± 1.33
C4-2B XRCC1-KO C-1	0.15 ± 0.01	0.12 ± 0.00	not determined
C4-2B XRCC1-KO C-2	0.19 ± 0.03	0.12 ± 0.00	not determined
22RV1 vector	10.87 ± 1.89	2.48 ± 0.65	19.25 ± 4.11
22RV1 XRCC1-KO C-1	1.18 ± 0.09	0.13 ± 0.01	not determined
22RV1 XRCC1-KO C-2	1.19 ± 0.16	0.14 ± 0.01	not determined

We further confirmed the susceptibility of C4-2B and 22RV1 *XRCC1-*KO C-1 and C-2 to the PARPi by clonogenic assay. When exposed to the PARPi, all the XRCC1-KO clones experienced a substantial impairment in colony-forming ability (Supplementary Fig. S2A and B).

To confirm that XRCC1 loss is responsible for the observed hypersensitivity to PARPi, we stably rescued the *XRCC1* expression in C4-2B and 22RV1 *XRCC1-*KO C-1 and C-2 by transducing them with a PAM (protospacer adjacent modifier) sequence modified *XRCC1* CDS containing overexpression vector. The rescued *XRCC1* expression was confirmed in these clones by immunoblotting (Fig. 3C and D). We then checked the sensitivity of *XRCC1* rescued clones against a single dose of rucaparib, talazoparib, or olaparib by growth inhibition assay. The results showed that ectopic *XRCC1* expression reversed the hypersensitive phenotype of C4-2B and 22RV1 *XRCC1-*KO C-1 and C-2 (Fig. 3E and F). These data demonstrate that *XRCC1* deficiency confers sensitivity to PARPi in mCRPC cells. We also checked the growth of C4-2B and 22RV1 vector, *XRCC1-*KO C-1, *XRCC1-*KO C-1, *XRCC1-*KO C-1 + *XRCC1*, and *XRCC1-*KO C-1 + *XRCC1* clones in absence of any of these PARPi. *XRCC1* depletion alone decreased cell growth, which was reversed by the ectopic expression of *XRCC1* (Supplementary Fig. S3A and B).

### *XRCC1* depletion enhances PARPi-induced DNA damage in PCa cells

Next, we explored the mechanism by which *XRCC1* deficiency increased the cell growth inhibition by PARPi. Given the importance of XRCC1 in repairing SSBs [19], we hypothesized that XRCC1 loss resulted in the inhibition of DNA repair with the hypersensitive response of *XRCC1*-KO cells to PARPi due to pronounced DNA damage accumulation. As both *XRCC1-*KO C-1 and C2 displayed similar results in the above experiment, we limited our further experiments to only *XRCC1*-KO C-1 in both the C4-2B and 22RV1 cell lines.

**Figure 4.**
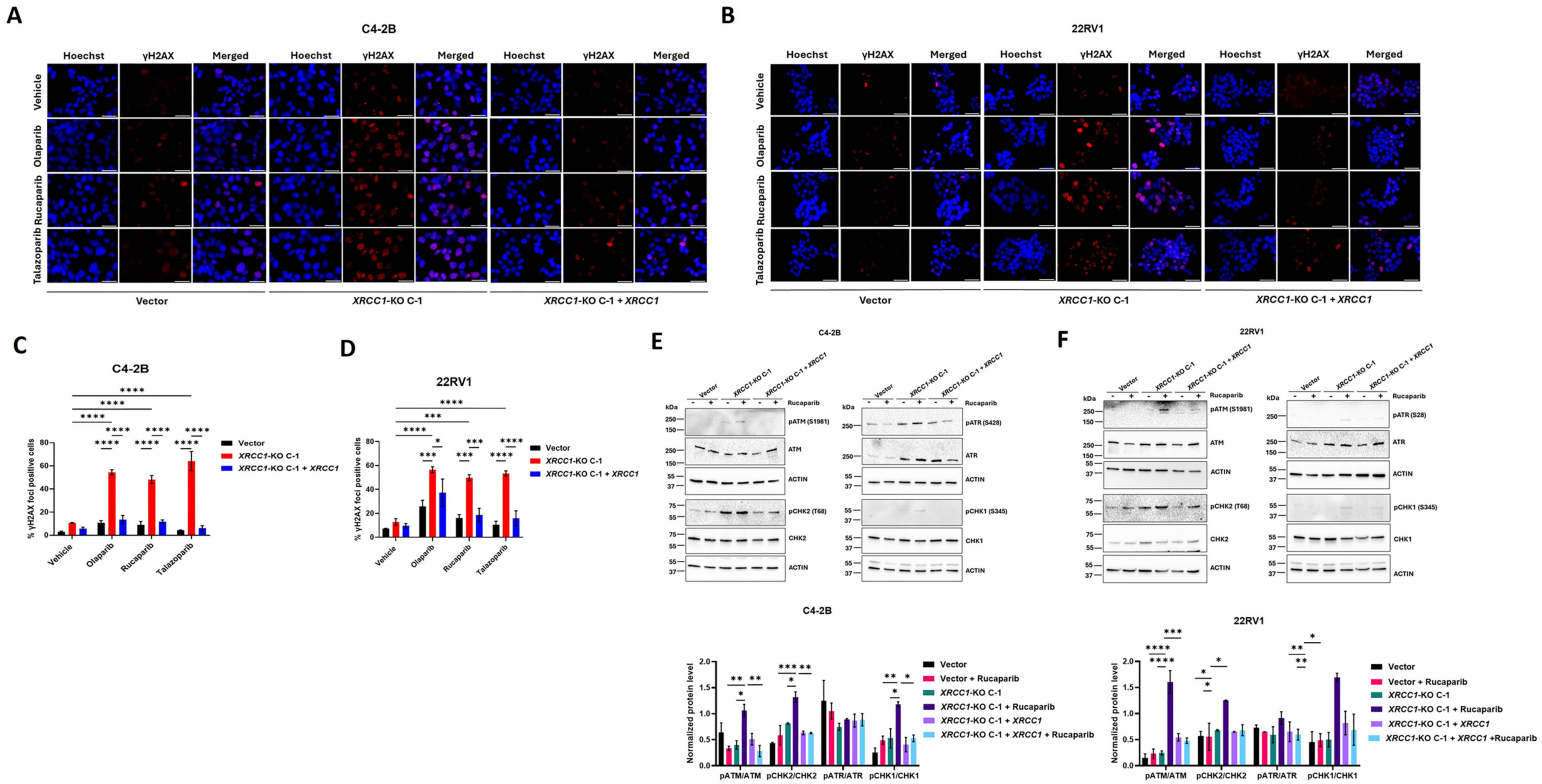
*XRCC1* knockout increased PARPi-induced DNA damage and damage response signaling in PCa cells. (**A** and **B**) Representative microscopy images of γ-H2AX foci positive C4-2B (A) and 22RV1 (B) vector control cells, *XRCC1-*KO C-1, and *XRCC1-*KO C-1 + *XRCC1* following 48 h treatment with PARPi. 0.25 μM (for C4-2B) and 2.5 μM (for 22RV1) olaparib, 0.5 μM rucaparib, and 0.5 nM talazoparib was used to dose the cells. The nucleus was visualized by Hoechst 33 342. Scale bar: 50 μm. (**C** and **D**) Quantification of percent γH2AX foci positive cells in the same cells as in panels (A) and (B). (**E** and **F**) Immunoblots (upper) and quantification (lower) show the expression of phosphorylated and total level of DDR signaling proteins after 48 h treatment with 0.5 μM rucaparib only. Actin was used as a loading control. The level of statistical significance among the groups was computed using two-way ANOVA with Tukey’s multiple comparisons test. The level of statistical significance is indicated as follows: *P < 0.05, **P < 0.01, ***P < 0.001, and ****P < 0.0001.

Using immunofluorescence, we measured the formation of phosphorylated histone H2AX (γH2AX) foci, which signal SSBs and DSBs (Kopp *et al.*). Forty-eight hours post PARPi treatment, C4-2B and 22RV1 *XRCC1-*KO C-1 exhibited a significantly higher percentage of cells with γH2AX foci compared with PARPi-treated respective vector controls (Fig. 4A–D). Ectopic expression of *XRCC1* in C4-2B and 22RV1 *XRCC1-*KO C-1 cells decreased the PARPi-induced γH2AX foci levels (Fig. 4A–D). We also observed that *XRCC1* depletion alone increased the percentage of γH2AX foci-positive cells, but the effect was even more significantly enhanced in *XRCC1-*KO C-1 PARPi-treated cells. This data suggests that *XRCC1* loss sensitizes the cells to PARPi treatment, inducing increased amounts of DNA damage.

DNA replication stress and DSBs activate ATR and ATM proteins, prompting a cascade of DNA damage response (DDR) signaling via their kinase activities. This DDR signaling regulates the cell cycle checkpoints and apoptosis or repair DNA damage to maintain the genomic integrity of cells [29]. To confirm the increase in DNA DSBs, we performed immunoblotting to examine the activation of ATR-CHK1 and ATM-CHK2 signaling axis in C4-2B and 22RV1 *XRCC1-*KO C-1 following 48 h of rucaparib treatment. From here onwards, we limited the experiments only to rucaparib due to two reasons; first, in our cell growth inhibition assay, rucaparib was found to decrease the cell survival more efficiently as compared to the olaparib (20–30-fold decrease in IC_50_ value in case of rucaparib in C4-2B and 22RV1 respectively as compared to the 10-fold decrease in case of olaparib) and second, IC_50_ value of rucaparib was almost similar in both the C4-2B and 22RV1 vector control cell lines. We did not proceed with talazoparib due to its high toxicity in *XRCC1*-KO C-1 cells, making it difficult to grow for further experimentation.

The results showed that the C4-2B and 22RV1 *XRCC1-*KO C-1 contain elevated levels of phosphorylated ATM to total ATM (pATM/ATM), which increased after rucaparib treatment (Fig. 4E and F). However, the levels of phosphorylated ATR to total ATR (pATR/ATR) were unchanged after rucaparib treatment in vector control cells or *XRCC1-*KO cells (Fig. 4E and F). In addition, we found that the levels of phosphorylated CHK2 to total CHK2 (pCHK2/CHK2) and phosphorylated CHK1 to total CHK1 (pCHK1/CHK1), the downstream targets of ATM and ATR, were increased in both the C4-2B and 22RV1 *XRCC1-*KO C-1 upon rucaparib treatment (Fig. 4E and F). Ectopic expression of *XRCC1* in the C4-2B and 22RV1 *XRCC1-*KO C-1 cells decreased the levels of PARPi-induced DNA damage signaling proteins in both the C4-2B and 22RV1 *XRCC1-*KO C-1, demonstrating rescue of the XRCC1-mediated effects (Fig. 4E and F). We also checked the XRCC1 expression level after 48 h of rucaparib treatment in C4-2B and 22RV1 wild-type cells to determine whether PARPi treatment induces XRCC1 expression. Interestingly, rucaparib treatment didn’t alter the XRCC1 protein level in either the C4-2B and 22RV1 cells (Supplementary Fig. S4A and B).

### *XRCC1* depletion enhances PARPi-induced chromatin-associated PARP1 in PCa cells

**Figure 5.**
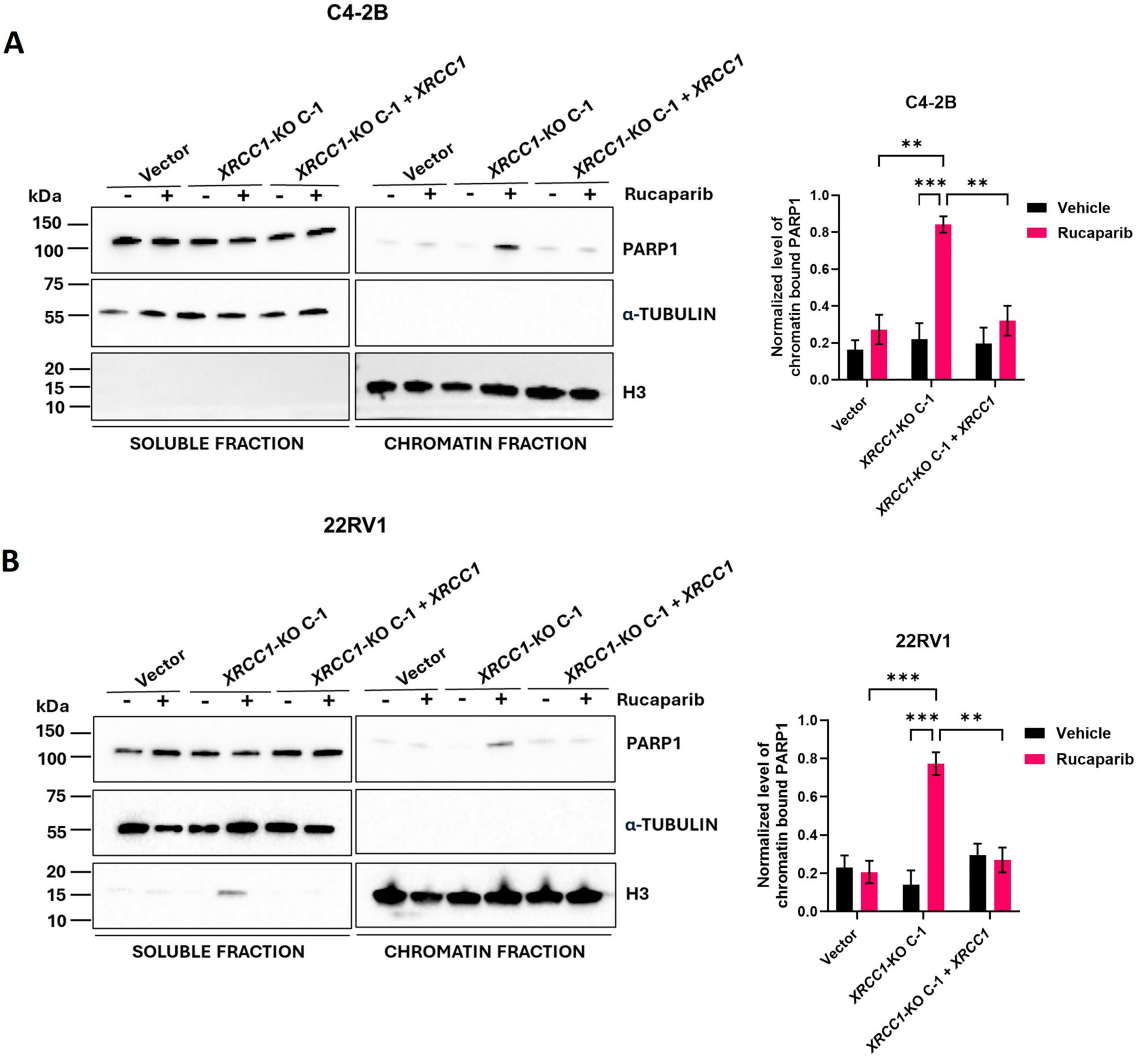
*XRCC1* knockout increased PARPi-induced chromatin-associated PARP1 in PCa cells. (**A** and **B**) Immunoblots (left) and quantification (right) show the expression of PARP1 in soluble and chromatin fractions of C4-2B (A) and 22RV1 (B) vector control cells, *XRCC1-*KO C-1, and *XRCC1-*KO C-1 + *XRCC1* following 24 h treatment with rucaparib only. α-Tubulin and H3 were used as a loading control for soluble and chromatin fractions, respectively. The level of statistical significance among the groups was computed using two-way ANOVA with Tukey’s multiple comparisons test. The level of statistical significance is indicated as follows: **P < 0.01 and ***P < 0.001.

In addition to PARP1 catalytic inhibition, PARP inhibition can induce trapping or crosslinking of PARP1 onto the DNA. The trapped PARP1–DNA complexes block the replication fork progression and result in the generation of DSBs [7, 30]. Since XRCC1 deficiency enhanced PARPi-induced DNA damage (Fig. 4), we next evaluated whether XRCC1 deficiency impacted chromatin-associated PARP1 as an indicator of trapped PARP1–DNA, following rucaparib treatment by performing chromatin fractionation [7, 8]. Rucaparib-treated C4-2B and 22RV1 *XRCC1-*KO C-1 contained elevated levels of chromatin-associated PARP1 compared to the rucaparib-treated vector control cells or *XRCC1-*KO untreated cells (Fig. 5A and B). This increase in chromatin-associated PARP1 was reversed upon ectopic expression of *XRCC1* in rucaparib-treated C4-2B and 22RV1 *XRCC1-*KO C-1 cells.

### *XRCC1* depletion enhances PARPi-induced cell cycle arrest in PCa cells

**Figure 6.**
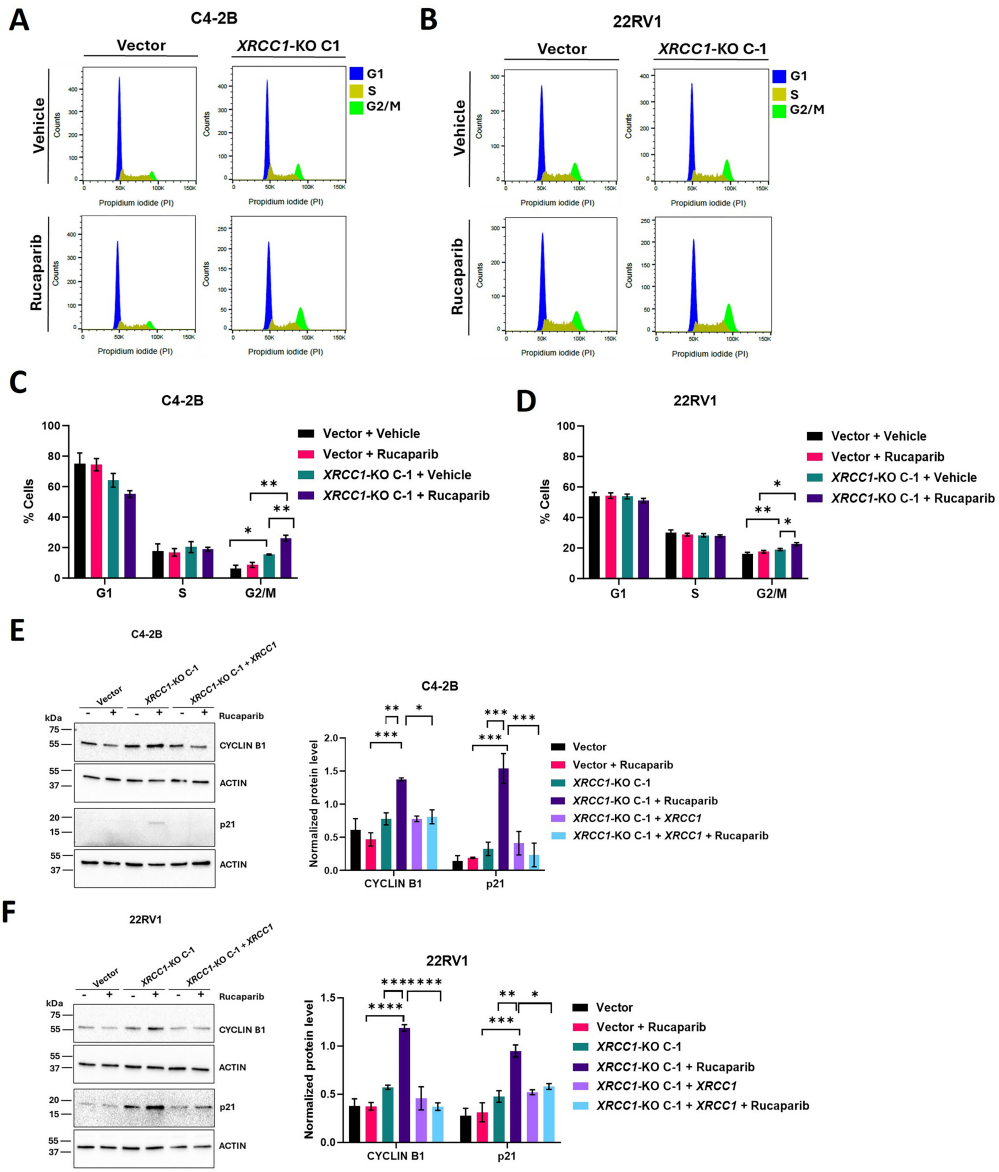
*XRCC1* depletion enhanced PARPi-induced cell cycle arrest. (**A** and **B**) Representative cell cycle profiles of C4-2B (A) and 22RV1 (B) vector control cells and *XRCC1-*KO C-1 following 48 h treatment with 0.5 μM rucaparib using PI DNA staining-based flow cytometry. (**C** and **D**) Percent distribution of cells in G1, S, and G2/M phase of the cell cycle. (**E** and **F**) Immunoblots (left) and quantification (right) show the expression of cell cycle arrest-related proteins in C4-2B (E) and 22RV1 (F) vector control cells, *XRCC1-*KO C-1, and *XRCC1-*KO C-1 + *XRCC1* after 48 h treatment with 0.5 μM rucaparib. Actin was used as a loading control. The level of statistical significance among the groups was computed using two-way ANOVA with Tukey’s multiple comparisons test. The level of statistical significance is indicated as follows: *P < 0.05, **P < 0.01, and ***P < 0.001.

Since we observed a prominent increase in DNA DSBs and DDR signaling in *XRCC1-*KO cells upon treatment with rucaparib, we evaluated whether *XRCC1* deficiency impacted cell cycle progression following rucaparib treatment using the flow cytometry. Forty-eight hours post-treatment, rucaparib led to a significant increase in the accumulation of cells in the G2/M phase in *XRCC1-*KO C-1 (26.15 ± 1.93%; *P =*0.002 in C4-2B and 22.53 ± 1.00%; *P =*0.001 in 22RV1) as compared to their respective rucaparib-treated vector control cells (8.63 ± 1.66% in C4-2B and 17.50 ± 0.87% in 22RV1) (Fig. 6A–D). Consistent with the increased DNA damage in C4-2B and 22RV1 *XRCC1-*KO C-1 cells (Fig. 6A–D), their population in G2/M phase was also increased (15.50 ± 0.26%; *P*= 0.001 in C4-2B and 19 ± 0.70%; *P*= 0.003 in 22RV1) compared to vector control cells (6.11 ± 2.33% in C4-2B and 16.13 ± 1.00% in 22RV1). The effect was even more significantly pronounced in *XRCC1-*KO C-1 rucaparib-treated cells. Moreover, the rucaparib-treated vector control cells didn’t show any significant differences in G2/M phase (8.63 ± 1.66%; *P*= 0.31 in C4-2B and 17.50 ± 0.87%; *P*= 0.13 in 22RV1) as compared to untreated vector control cells (6.11 ± 2.33% in C4-2B and 16.13 ± 1.00% in 22RV1).

We further confirmed the accumulation of rucaparib-treated C4-2B and 22RV1 *XRCC1*-KO C-1 cells in G2/M phase by examining the expression levels of p21 (Fig. 6E and F). p21 is one of the main targets of ATM and ATR DDR signaling, and it regulates G2/M checkpoint activation and maintenance [31]. Rucaparib-treated C4-2B and 22RV1 *XRCC1*-KO C-1 showed robust expression of p21 compared to their respective rucaparib-treated vector control cells. Similar to the cell cycle profiles observed, we detected a minor upregulation of p21 in C4-2B and 22RV1 *XRCC1*-KO C-1 compared to their vector control cells. We also checked the level of cyclin B1 protein, which peaks at the G2/M transition [32]. Consistent with the p21 upregulation, the rucaparib-treated C4-2B and 22RV1 *XRCC1*-KO C-1 cells showed the highest accumulation of cyclin B1 following rucaparib treatment (Fig. 6E and F). Ectopic expression of *XRCC1* in the C4-2B and 22RV1 *XRCC1-*KO C-1 cells decreased the levels of PARPi-induced cyclin B1 and p21, indicating rescue (Fig. 6E and F).

### *XRCC1* depletion enhances PARPi-induced apoptosis in PCa cells

**Figure 7.**
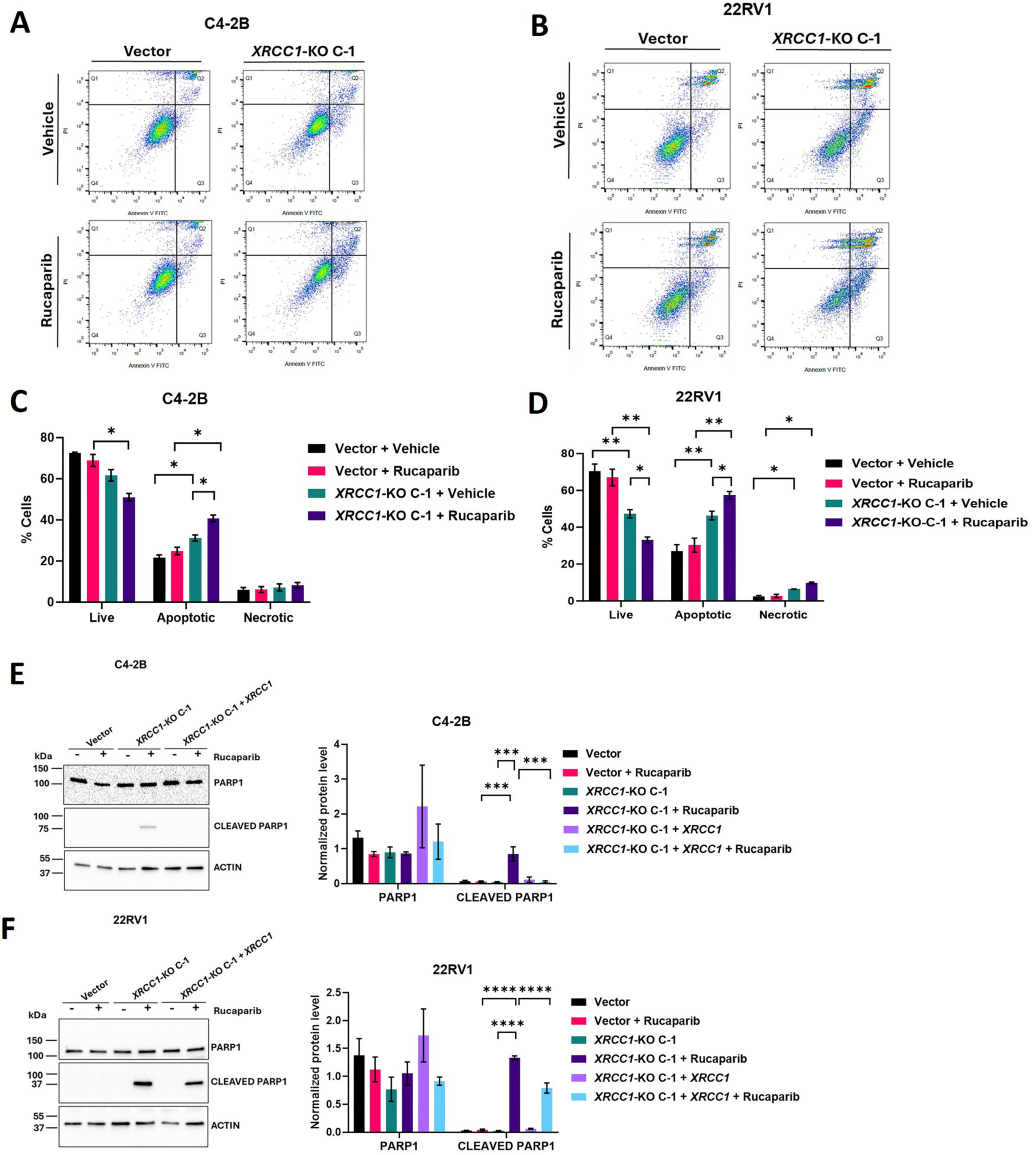
*XRCC1* depletion enhanced PARPi-induced apoptosis. (**A** and **B**) Representative apoptosis profiles of C4-2B (A) and 22RV1 (B) vector control cells and *XRCC1-*KO C-1 following 48 h treatment with 0.5 μM rucaparib using annexin V-PI dual staining-based flow cytometry. (**C** and **D**) The percent apoptotic population is indicated. (**E** and **F**) Immunoblots (left) and quantification (right) show the expression of cleaved and total PARP-1 in C4-2B (E) and 22RV1 (F) vector control cells, *XRCC1-*KO C-1, and *XRCC1-*KO C-1 + *XRCC1* after 48 h treatment with 0.5 μM rucaparib. Actin was used as a loading control. The level of statistical significance among the groups was computed using two-way ANOVA with Tukey’s multiple comparisons test. The level of statistical significance is indicated as follows: *P < 0.05, **P < 0.01, ***P < 0.001, and ****P < 0.0001.

We next determined whether the observed decrease in cell survival in PARPi-treated *XRCC1*-KO cells was due to apoptosis-mediated cell death. We used Annexin V-FITC/PI dual staining and flow cytometric analysis. Annexin-V+*/*PI− (Q3 quadrant) and Annexin-V+*/*PI+ (Q2 quadrant) represented early apoptotic cells and late apoptotic cells, respectively, and apoptotic cells were counted as both late and early apoptotic cells. Annexin-V−*/*PI+ (Q1 quadrant) and Annexin-V−*/*PI− (Q4 quadrant) were considered to represent necrotic cells and living cells, respectively. After 48 h, rucaparib led to a dramatic increase in the percentage of apoptotic events in *XRCC1-KO* C-1 (40.72 ± 1.66%; *P =*0.02 in C4-2B and 57.34 ± 1.99%; *P =*0.003 in 22RV1) as compared to their respective rucaparib-treated vector control cells (24.92 ± 1.79% in C4-2B and 30.28 ± 3.84% in 22RV1) (Fig. 7A–D). A small, nonsignificant increase in the percentage of necrotic events in rucaparib-treated C4-2B and 22RV1 *XRCC1-KO* C-1 cells was also observed. We observed increased apoptosis in *XRCC1*-KO C-1 (31.19 ± 1.50%; *P =*0.02 in C4-2B and 46.31 ± 2.36%; *P =*0.008 in 22RV1) compared to vector control cells (21.72 ± 1.23% in C4-2B and 27.11 ± 3.49% in 22RV1), but the increase was more pronounced in rucaparib-treated *XRCC1-*KO C-1 cells. The rucaparib-treated vector control cells did not show a significant increase in the percentage of apoptotic population (24.92 ± 1.79%; *P*= 0.20 in C4-2B and 30.28 ± 3.84%; *P*= 0.23 in 22RV1) as compared to untreated vector control cells (21.72 ± 1.23% in C4-2B and 27.11 ± 3.49% in 22RV1).

We validated the increased apoptotic nature of cell death in rucaparib-treated C4-2B and 22RV1 *XRCC1*-KO C-1 by checking the levels of cleaved PARP1, which is a classic marker of apoptosis [33, 34]. Indeed, a highly elevated level of cleaved PARP-1 was detected after 48 h of the rucaparib treatment in both the C4-2B and 22RV1 *XRCC1*-KO C-1 compared with their respective rucaparib-treated vector control cells (Fig. 7E and F). Ectopic expression of *XRCC1* in the C4-2B and 22RV1 *XRCC1-*KO C-1 cells decreased the levels of PARPi-induced cleaved PARP1, indicating rescue (Fig. 7E and F).

### *XRCC1* level dictates the response of PCa cells towards PARPi

**Figure 8.**
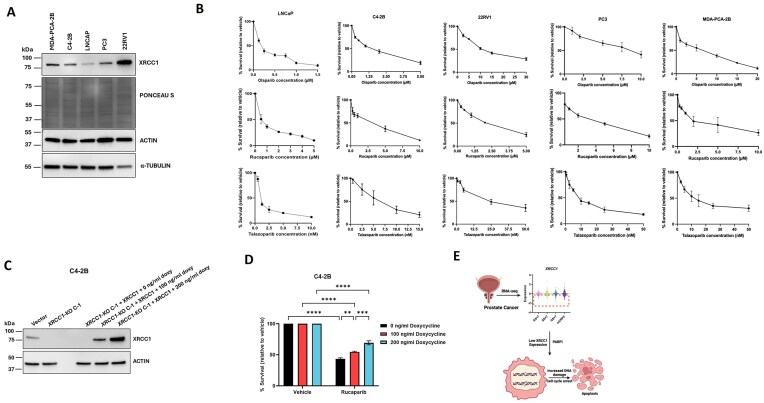
*XRCC1* expression level dictates the response to PARPi in PCa cells. (**A**) Immunoblot shows the expression of XRCC1 protein in different PCa cell models, including MDA-PCA-2B, C4-2B, LNCaP, PC3, and 22RV1. Ponceau Stain was used as a loading control. (**B**) Cell growth inhibition assay for olaparib (upper), rucaparib (middle), and talazoparib (lower) in LNCaP, MDA-PCA-2B, C4-2B, PC3, and 22RV1. The cells were incubated in the drug-containing media for 6 days. (**C**) Immunoblot shows the expression of XRCC1 protein in C4-2B doxycycline-inducible *XRCC1* CDS-overexpressing *XRCC1-*KO C-1 C4-2B cells following 24 h treatment with indicated dose of doxycycline. (**D**) The survival percentage of these cells was calculated after treatment with 10 μM rucaparib for 72 h. The level of statistical significance among the groups was computed using two-way ANOVA with Tukey’s multiple comparisons test. The level of statistical significance is indicated as follows: **P < 0.01, ***P < 0.001, and ****P < 0.0001. (**E**) Model for how the expression of XRCC1 may be used as a stratification method for PARPi use in PCa. Created in BioRender (Gassman, N. (2025); https://BioRender.com/ne48py9).

The above findings suggest that complete *XRCC1* loss confers sensitivity towards PARPi. We were next interested in determining if there is any correlation between the levels of *XRCC1* and PARPi activity. To determine this, we first examined the XRCC1 protein level in five different PCa cell lines, namely, C4-2B, 22RV1, PC3, LNCaP, and MDA-PCA-2B. The XRCC1 protein level was found to be the highest in 22RV1 > MDA-PCA-2B > PC3 ≥ C4-2B > LNCaP (Fig. 8A). We used Ponceau Stain as a loading control for the normalization of XRCC1 blot since we were not able to get either actin or tubulin as a consistent loading control across the different cell lines (Fig. 8A).

We then performed a cell growth inhibition assay for LNCaP, PC3, and MDA-PCA-2B cell lines for the three different PARPi (olaparib, rucaparib, and talazoparib) and calculated the IC_50_ value for each of them (Fig. 8B and Table 3). For the C4-2B and 22RV1, we used the already calculated IC_50_ value (Fig. 3A and B and Table 2). Indeed, a strong direct correlation between the IC_50_ value of olaparib or talazoparib and XRCC1 was found in these cells as the IC_50_ value of both followed the same order in PCa cells as of XRCC1 expression level (Fig. 8A). In the case of rucaparib, this correlation was missing and all the cell lines except LNCaP showed almost a similar IC_50_ value, which may indicate other mechanism action differences with this drug across the different PCa backgrounds.

**Table 3.** IC_50_ values of PARPi in different PCa parental cell lines

Cell line	Olaparib IC_50_ (μM)	Rucaparib IC_50_ (μM)	TalazoparibIC_50_ (nM)
**LNCaP**	0.14± 0.03	0.47± 0.08	0.73± 0.09
**C4-2B**	1.35 ± 0.23	3.01 ± 0.17	5.43 ± 1.33
**PC3**	4.12 ± 0.82	2.60 μM ± 0.23	8.45 ± 1.90
**MDA-PCA-2B**	7.39 ± 1.63	1.8 ± 0.33	8.61 ± 2.91
**22RV1**	10.87 ± 1.89	2.48 ± 0.65	19.25 ± 4.11

Next, we generated doxycycline-inducible XRCC1 CDS-overexpressing *XRCC1*-KO C-1 C4-2B cells. We then treated these cells with two different increasing concentrations of doxycycline for 24 h and confirmed the gradual increase of XRCC1 protein level (Fig. 8C). We performed a cell growth inhibition assay at the same concentrations of doxycycline by treating the cells with rucaparib. Cell survival increased gradually, corresponding to an increase in doxycycline concentration or the XRCC1 level (Fig. 8D). Figure 8E presents a theoretical model for how the expression of XRCC1 could be used as a biomarker to stratify patients for PARPi use given the dose-response relationship observed in Fig. 8A–D.

## Discussion

Higher metastatic potential, enhanced biochemical recurrence, and poor patient survival are features of advanced stage PCa [35]. Different PARPi, such as olaparib, rucaparib, and talazoparib, have shown promising results either alone or in combination with other therapies in the clinical management of multiple cancers, including PCa tumors harboring defects in HRR [9, 10, 36]. However, the low percentage of patients showing defects in HR genes limits the application of PARPi therapy on a wider scale in PCa. In the case of mCRPC, the percentage of patients showing HR defects is <20% [37]. Therefore, it is necessary to determine alternative genetic and epigenetic biomarkers that may sensitize PCa tumors to PARPi.

Several studies have demonstrated the potential of BER proteins such as XRCC1 and POL β in sensitizing cells towards cell death by PARPi [12–14]. XRCC1 is a scaffold protein coordinating and facilitating downstream BER or SSBR factors [38, 39]. Several studies have shown the effectiveness of exploiting *XRCC1* deficiency to enhance the therapeutic effects of DNA damaging cancer therapies, considering its important role in DNA repair. XRCC1 loss amplifies the sensitivity of pancreatic cancer cells to β-lapachone [40], triple negative breast cancers to the ATM, ATR, and Wee1 inhibitors [41], liver adenocarcinoma HepG2 to γ rays [42], and breast cancer cells to cisplatin, camptothecin, and MMS [43]. Most interestingly, XRCC1 depletion sensitizes ovarian and breast cancer preclinical models to PARPi [16, 44].

In examining the levels of *XRCC1* using two different PCa datasets, XRCC1 expression varies widely across tumors across stages and Gleason scores. Thus, low expression of *XRCC1* may serve as a relevant clinical biomarker for treating PCa patients of all stages with PARPi. We validated the potential use of low XRCC1 expression as a biomarker for PARPi use by demonstrating that XRCC1 loss enhances PCa cell killing by olaparib, rucaparib, and talazoparib compared to the *XRCC1* proficient cell lines (Fig. 3). These data expand the findings by Tsujino *et al.* who found *XRCC1* knockdown as a determinant of olaparib sensitivity in multiple PCa cell models [45]. We also expanded on this work to show a correlation between XRCC1 protein expression and sensitivity to olaparib and talazoparib (Table 3). Higher expression showed increased resistance to PARPi. We also examined the Genomics of Drug Sensitivity in Cancer database (https://www.cancerrxgene.org/) and found the LNCaP to show the highest sensitivity to these PARPi, similar to our results and consistent with the low expression of XRCC1. We could not perform a complete analysis of all the cell lines used in this work with this database since not all cell lines were tested in the database, and some cell lines were only tested with olaparib and not rucaparib or talazoparib. Together, these findings support PCa cells’ differing sensitivity to PARPi and the fact that XRCC1 can act as a biomarker for PARPi response.

Another interesting finding in this work is that rucaparib sensitivity differed from olaparib and talazoparib, suggesting other cellular interactions contribute to cell killing with this agent. Our extensive testing of rucaparib and its mechanism of cell killing in this work show that XRCC1 loss enhances the impact of rucaparib despite different cell backgrounds (Figs 3–7). *XRCC1-*KO cells display enhanced DNA damage, ATM DDR signaling pathways activation, and PARP1 chromatin trapping. Ectopic expression of *XRCC1* rescued these effects, demonstrating that the loss of XRCC1 mediates them. Rucaparib sensitivity has never been examined earlier in the literature in combination with XRCC1 deficiency.

The elevated DNA damage further increased rucaparib-induced G2/M phase cell cycle arrest and apoptosis in *XRCC1-*KO cells. Similar to our findings, a previous study reported that rucaparib treatment of glioblastoma cells arrested the cell cycle in the G2/M phase [46]. Also, olaparib treatment to gastric cells led to increased G2/M phase cell cycle arrest [47]. 48 h after rucaparib treatment, the apoptotic population in ovarian cancer cells increased significantly [48]. In addition, rucaparib treatment to atypical teratoid rhabdoid tumor cell lines led to increased apoptosis and activity of caspase-3 in a dose-dependent manner [49]. Our results showed increased rucaparib-induced apoptosis correlated with these earlier studies and, more importantly, were dependent on XRCC1 expression in the PCa models.

PARP1 protein recognizes and binds to the SSBs generated by endogenous and exogenous agents. It PARylates other repair proteins and itself to recruit factors to the DNA damage site and eventually dissociates itself and them upon repair [50]. PARPi inhibits the catalytic activity of PARPi and, thus, the PARP1 autoPARylation. It also leads to the trapping or crosslinking of PARP1 onto the DNA. The trapped DNA–PARP1 complexes block the replication fork progression and result in the generation of DSBs [7, 30]. A study published in 2021 suggests that increased PARP1 engagement also causes the accumulation of SSBs in XRCC1^−/−^ cells, assuming XRCC1 deficiency leads to SSBs [28]. The authors concluded that XRCC1 acts as an endogenous anti-trapper for PARP1 and, in the absence of XRCC1, PARP1 binds to DNA similarly to its treatment with PARPi. This conclusion was also supported by another study by Hirota *et al.*, where XRCC1 prevented the PARP1 trapping caused by olaparib [51]. The increased cell killing and DNA damage by PARPi in our *XRCC1* deficient cell lines is also attributed to this mechanism of action. The *XRCC1-*KO cells display enhanced chromatin-associated PARP1, i.e. trapped PARP1, upon PARPi treatment. The restoration of XRCC1 expression reverses these effects (Fig. 5).

The synergistic interaction of XRCC1 deficiency and PARPi in PCa is further validated by our observations in *XRCC1*-KO cells, where increased strand breaks are observed after XRCC1 loss, and these breaks are further enhanced with the addition of rucaparib. Additionally, the extreme cytotoxicity of talazoparib (a more potent PARP1 trapper) compared to olaparib and rucaparib in the *XRCC1*-KO cells is consistent with these two mechanisms operating in concert. While we cannot eliminate other mechanisms of PARPi toxicity, such as catalytic inhibition, reactive oxygen species (ROS) generation, and transcriptional machinery modulation, our results strongly support increased DNA damage due to increased chromatin-associated PARP1 as a mechanism of action when XRCC1 levels are low.

Collectively, these results highlight that XRCC1 levels are a potential biomarker for PARPi use in PCa. The IC_50_ value of olaparib and talazoparib in different PCa cell models with varying XRCC1 protein levels and our doxycycline-inducible system provides evidence of a tight inverse correlation between *XRCC1* expression level and PARPi sensitivity. Therefore, the complete XRCC1 depletion or its low expression level can increase PARPi sensitivity in PCa. Since we showed in our previous study that AA men have particularly low XRCC1 protein levels [22], we propose using XRCC1 expression levels to stratify these patients for PARPi use, which could improve clinical outcomes.

Current PARPi trials in PCa have not examined the gene expression of XRCC1 or other BER proteins, which may enhance PARPi sensitivity. Expanding criteria for PARPi use beyond mutations in HR genes would offer broader opportunities to exploit DNA repair defects and improve care of this complex disease. The next critical step is developing benchmarks for gene or protein expression in PCa patients for PARPi use. A similar strategy was reported with *SLFN11* in small cell lung cancer. The authors of the study showed that *SLFN11* expression level, as depicted by immunohistochemistry (IHC), is associated with tumor response to talazoparib in multiple patient-derived xenograft models [52]. Future studies are required to translate XRCC1 mRNA expression or IHC into the clinical setting to validate our preclinical findings. However, this work establishes the potential to use XRCC1 deficiency as an indicator for PARPi use and suggests that PARPi selection is also an important factor for use in PCa.

## Supplementary Material

zcaf015_Supplemental_File

## Data Availability

The data underlying this article are available in figshare at https://doi.org/10.6084/m9.figshare.28716008.
