# Supplementary material for: PARP inhibitor response is enhanced in prostate cancer when XRCC1 expression is reduced
Source: NAR Cancer. 2025 Apr 23;7(2):zcaf015. doi: 10.1093/narcan/zcaf015 (PMC12015684; doi:10.1093/narcan/zcaf015)
Supplement: zcaf015_Supplemental_File [file zcaf015_supplemental_file.pdf]

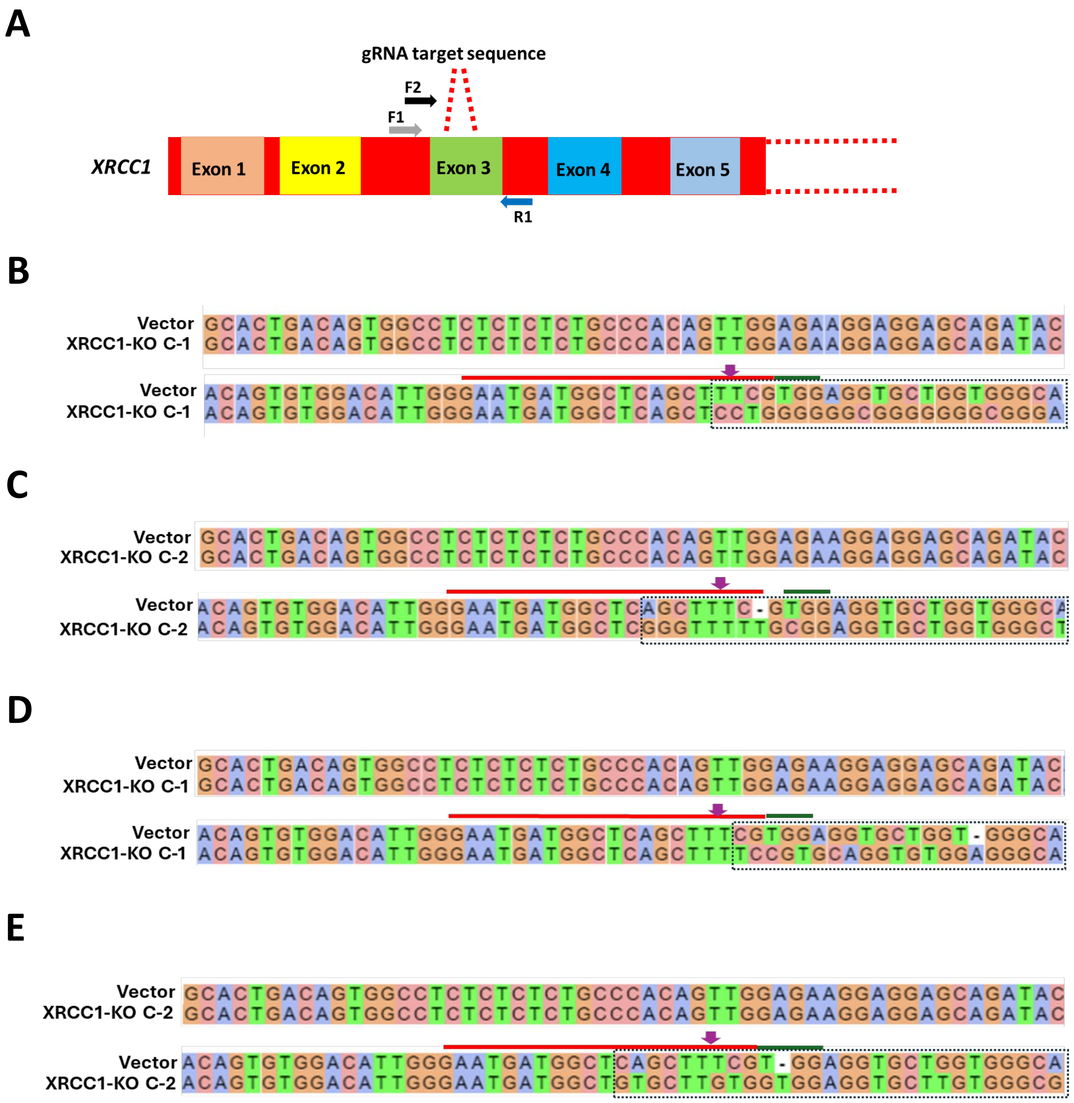

Supplementary Figure 1: *XRCC1* structure and nucleotide sequence alignment between vector and *XRCC1*-KO cell lines. (A) The structure of the XRCC1 gene with the gRNA targeting sequence in exon 3 and the location of primers used for amplification and sequencing of the region surrounding the gRNA target sequence. Primer pairs F1 and R1 were used for PCR amplification, and primer F2 was used to sequence the obtained amplicon. (B-E) Multiple sequence alignment pictures of the (B) C4-2B vector and *XRCC1*-KO C-1, (C) C4-2B vector and *XRCC1*-KO C-2, (D) 22RV1 vector and *XRCC1*-KO C-1, and (E) 22RV1 vector and *XRCC1*-KO C-2. In Fig. B-E, regions with red lines represent the gRNA target sequence, regions with dark green lines represent the PAM sequence, the blue arrows point towards the Cas9 cut site, and the regions enclosed in dashed line boxes represent unaligned nucleotides of the *XRCC1* gene between vector control and *XRCC1*-KO cell lines.

A

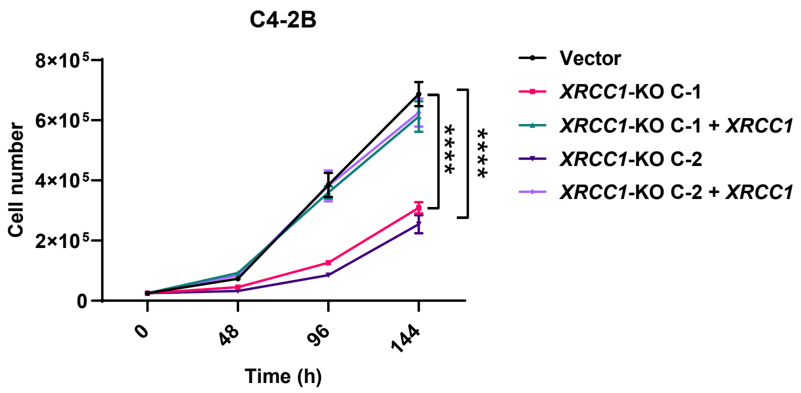

B

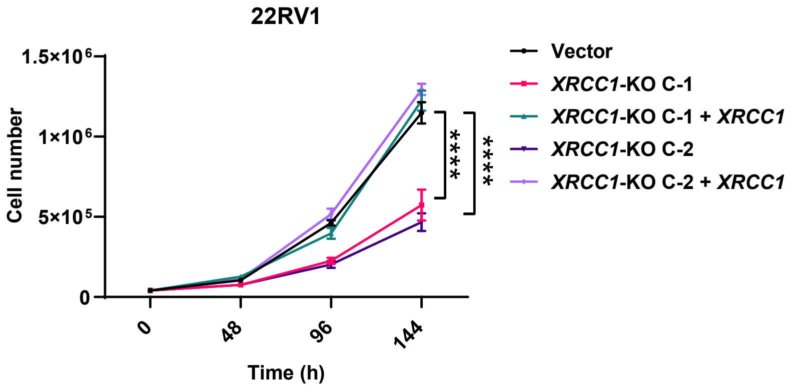

Supplementary Figure 2: *XRCC1* knockout reduces the growth of PCa cells. (A and B) Number of C4-2B (A) and 22RV1 (B) vector control, *XRCC1* KO-C1, *XRCC1*-KO C-1 + *XRCC1*, *XRCC1* KO-C2, and *XRCC1*-KO C-2 + *XRCC1* cells at different time points. The level of statistical significance was computed for the last timepoint using two-way ANOVA with Tukey's multiple comparisons test. The level of statistical significance is indicated as follows: ****p < 0.0001.

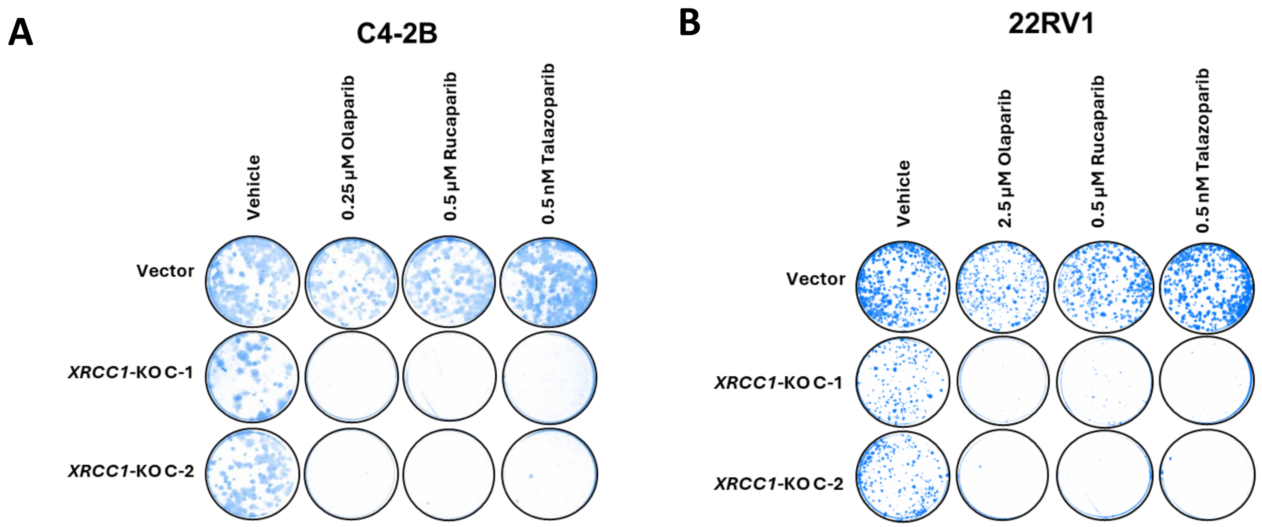

Supplementary Figure 3: *XRCC1* knockout enhanced sensitivity to PARP inhibitors in PCa cells. (A and B) Colony formation assay in C4-2B (A) and 22RV1 (B) vector control, *XRCC1* KO-C1, and *XRCC1* KO-C2 cells upon treatment with PARPi olaparib, rucaparib, and talazoparib. 0.25 µM (for C4-2B) and 2.5 µM (for 22RV1) olaparib, 0.5 µM rucaparib, and 0.5 nM talazoparib were used to dose the cells and the cells were kept in the same drug-containing media for 15 days.

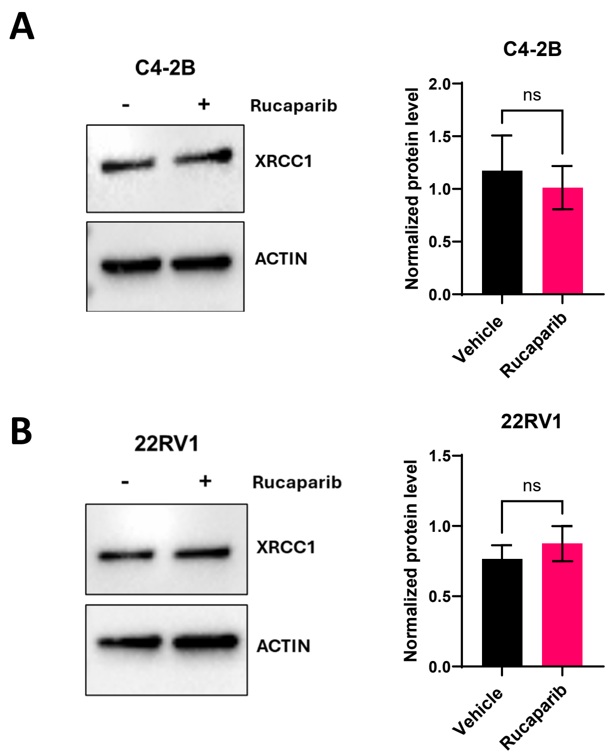

Supplementary Figure 4: Rucaparib treatment doesn't alter XRCC1 protein levels in PCa cells. (A and B) Immunoblots (left) and quantification (right) show the expression of XRCC1 in C4-2B (A) and 22RV1 (B) after 48 h treatment with 0.5 μM rucaparib. The level of statistical significance was computed using unpaired t test. The ns represents non-significant.
